# A Comparative Study of Nutritional Status, Knowledge Attitude and Practices (KAP) and Dietary Intake between International and Chinese Students in Nanjing, China

**DOI:** 10.3390/ijerph15091910

**Published:** 2018-09-03

**Authors:** Ijaz ul Haq, Zahula Mariyam, Min Li, Xiaojia Huang, Pan Jiang, Falak Zeb, Xiaoyue Wu, Qing Feng, Ming Zhou

**Affiliations:** 1 Department of Nutrition and Food Hygiene, School of Public Health, Nanjing Medical University, Longmian Avenue 101, Nanjing 211166, China; ijaz@njmu.edu.cn (I.u.H.); mariyamzahul@gmail.com (Z.M.); jppanpan@njmu.edu.cn (P.J.); falak106@gmail.com (F.Z.); xiaoyuewu@njmu.edu.cn (X.W.); 2 School of International Education, Nanjing Medical University, Longmian Avenue 101, Nanjing 211166, China; minli@njmu.edu.cn (M.L.); shawn2006@163.com (X.H.)

**Keywords:** international students, Chinese students, percent body fats, KAP, dietary intake

## Abstract

University students tend to have poor dietary practices, which ultimately affect their nutritional status. International students are becoming more prevalent in China. The current study aimed to compare the nutritional status, knowledge attitude and practices (KAP) and dietary intake between international and Chinese students in China. A comparative study was conducted in undergraduate students of Nanjing Medical University aged 17–31 years including 308 international and 393 Chinese students. Data was collected by self-administered questionnaire. Body composition was detected by bioelectrical impedance analysis (BIA). Student t-test and chi square test were used for comparison. Linear regressions were used for association of nutritional status with determinants. The prevalence of overweight and obesity in international student was higher than Chinese students. International male and female students were having significantly (*p* < 0.05) high BMI and percent body fats than Chinese male and female students. Nutritional KAP scores of Chinese students was significantly (*p* < 0.05) higher than international students. As for diet consumption, daily milk consumption was high in international students while daily egg and weekly fish and meat consumption were found more in Chinese students. Fast foods and carbonated drinks weekly consumption was significantly (*p* < 0.001) high in international students. After adjusted for age, gender, education, sleeping duration and physical exercise, the inverse association of nutritional KAP with BMI remained significant. Our data indicate that international students had more percent body fats, less nutritional KAP scores and unhealthy dietary habits than Chinese students. Proper nutrition education and guidance for improving good habits and nutritional status is suggested for international students.

## 1. Introduction

Recently, nutrition related health problems, like diabetes, obesity, cardiovascular diseases, and cancer, are frequently diagnosed, and found to have significant impact on human health [1]. According to an estimate by World Health Organization, 80% of the aforementioned chronic disease burden is due to the lifestyle and dietary factors [2]. Thus, it is essential to improve life style and dietary intake by taking balanced and nutritionally healthy diet to overcome various adverse medical conditions [3].

Nutritional knowledge is one of the important factors for selection of healthy and nutritious diet [4]. Improper nutritional knowledge is one of the main causes for nutritional problems, which adversely affect dietary practices. Also, understanding of nutritional attitude and beliefs of the community are essential factors to improve healthy eating, effectively [5].

Dietary habits and nutritional status has been shown to have strong relationship with cardiovascular diseases biomarker in students [6]. Young people are usually prone to adopt unhealthy dietary habits. Poor dietary habits, such as skipping of breakfast, low intake of milk, fish, fruits and vegetables, and high intake of fast food, sweets, and sugar-sweetened beverages are present in young university students [7]. Students, living away from their homes consume fewer amounts of vegetables, fruits and meat [8].

A mobile population, consisting of international students, moves from their origin countries to carry on their higher studies in host countries [9]. Earlier, a study stated that migration may have negative impact on health status because of dissimilar environment [10]. One study reported that a majority of students who migrate to the United States (US) gain weight and also there is a gradual shift in the dietary patterns of international students to an American diet [11]. In the case of China, the number of international students has increased sharply over the past few years. According to a study, 397,635 international students came to China for the completion of their studies in 2015 [12]. Recently, China launched one road one belt policy; more international students are expected to come to China in education exchanges. Proper nutrition planning and guidance may be helpful for this population to maintain good health and nutritional status.

Because of the limited data, a study needs to target both international and local Chinese university students for determination of nutritional status, KAP and dietary intake. Up to our best knowledge, this is the first study which aims to associate and compare the nutritional status, KAP and dietary intake of international students with Chinese students in Nanjing, China.

## 2. Methods

### 2.1. Ethical Approval

The study was approved by ethical review board of Nanjing Medical University (NJMUIRB (2018)003).

### 2.2. Study Population and Inclusion Criteria

A comparative study was carried out by targeting international and Chinese undergraduate students at Nanjing Medical University. The data was collected between October 2017 and March 2018. Participation in the study was anonymous, confidential and voluntary. Data comprised 340 international students and 430 Chinese students, out of which 22 international and 37 Chinese students were excluded due to missing important information. A total of 701 medical students (308 international (43.9%) and 393 Chinese students (56.1%)) were included in the final analysis. An inclusion criterion was set for the international students as healthy individuals born outside from China and were staying in China as international students. Healthy individuals born in China having Chinese nationalities were considered as Chinese students.

### 2.3. Data Collection

#### 2.3.1. Pretesting of Questionnaire 

20 students for pre-testing of questionnaires were selected. Time duration for filling of questionnaire and anthropometric measurements was noted. Unnecessary questions were excluded. The questionnaire was written in English as well as in Chinese languages.

#### 2.3.2. Socio-Demographic Economic Characteristics

A self-administered questionnaire was used to obtain respondents socio-demographic information including age, sex, educational level, financial status, country, sleeping duration and physical exercise. In financial status categories, non-satisfactory indicates low income (struggling to meet their immediate needs), satisfactory indicates average income and very good indicates upper class (having more savings to spend on luxuries) [13]. 

#### 2.3.3. Anthropometry

Weight was measured on beam-scale to the nearest 0.1 kg without shoes while wearing minimum clothing. Height was measured to the nearest 0.1 cm with bare feet on a stadiometer. Body mass index was calculated as Kg/m^2^ and categorized into underweight (BMI < 18.5), normal (18.5–24.9), overweigh (25–29.9) and obese (≥30) by following international classification for BMI [14]. Percent body fats were measured by TANITA MC-780MA bioelectrical impedance analysis (Tanita Corp, Tokyo, Japan). Tanita BIA body fats ranges for healthy adults were used [15].

#### 2.3.4. Students’ Nutrition Knowledge, Attitude and Practices Scores

A nutritional KAP questionnaire with close ended questions was developed and tested prior to formal investigation. KAP was evaluated using a number of questions rather than a structured questionnaire. As in literature, previously, researchers also used individual questions for KAP rather than a structured questionnaire [16]. Nutritional knowledge consists of 6 questions in True/false format followed by two questions each for attitude and practice section, respectively. In total of 10 questions, correct answer was given 1 score. No negative scoring was given on false answer. Total score was summed up for every individual [17]. Maximum score was 10. The content of questionnaire was developed on the basis of a previous study [18] and validated by nutrition experts from the university. To find validity of KAP questionnaire, spearman correlation was used. Spearman correlation coefficient for each question score with totally score was more than 0.3 with *p* < 0.001. Moreover, the Cronbach’s Alpha coefficient was more than 0.6, which showed the reliability of KAP questionnaire. According to Moss et al. Cronbach’s Alpha coefficient more than 0.6 is acceptable [19].

#### 2.3.5. Dietary Assessment Tool 

The dietary consumption of the respondents was assessed by semi quantitative food frequency questionnaire (FFQ). The dietary intake assessment tool was created to include food groups that are important when studying dietary habits, which was based on the questionnaire, adopted and validated to other FFQs for university students with some modification [20,21]. The participants reported their dietary information of the commonly used food items mentioned in food frequency questionnaire during the past 1 year. The food frequency list contained cereals (wheat flour, rice, noodles etc.); meat and products (beef, pork, mutton, chicken, etc.); dairy (milk, cheese, yogurt, etc.), fast food (chips, pizza, burger, etc.); fruits (apple, banana, mango, etc.); vegetables (green leafy vegetables, cabbage/cauliflower, pumpkin etc.); drinks (carbonated drinks, tea, coffee, etc.), nuts and legumes. Frequency of each food was assessed by 6 categories: >1 time/day, 1 time/day, 3–6 times/week, 1–2 times/week, 1–3 times/month, never/seldom [22]. Furthermore, dietary intake between international and Chinese students was compared.

### 2.4. Statistical Analysis 

Data was analyzed by statistical package for social sciences (Version 21; SPSS Inc., Chicago, IL, USA). Descriptive data were expressed as n (%), mean ± SD and median (IQR). Independent samples *t*-tests were used for comparison of normally distributed continuous data, while Mann Whitney tests were used for not normally distributed continuous data. Chi square tests were executed for comparison of categorical variables. Multiple linear regressions were used to find association of nutritional status with determinants. A *p* < 0.05 was considered significant.

## 3. Results

### 3.1. Baseline Characteristics of International and Chinese Students

The baseline characteristics of international and Chinese students are presented in Table 1. The mean age of international students was significantly higher (*p <* 0.001) than Chinese students. As a whole, the percentage of females was higher than males. The maximum numbers of international students were from first year (28.9%) while in Chinese the maximum were from 2nd year (41.7%). The financial status of more international students (26%) was very good compared to Chinese students. Based on BMI classifications, the prevalence of overweight (16.1%) and obesity (9.0%) was higher in international students as compare to Chinese students. As for sleep duration, 35% of international student were having short sleep (<6 h/day) as compare to 5.6% of Chinese students. There were statistical differences regarding age, education, financial status, BMI and sleeping time (*p* < 0.05). The description of students according to countries has been shown in Supplementary Materials Table S1.

### 3.2. Comparison of Anthropometric and Life Style Variables According to Gender in International and Chinese Students

The mean percent body fats of international males were significantly higher (*p* < 0.001) than international female students and Chinese male was higher than Chinese females’ students (Table 2). Also, the mean percent body fats of international male and female students were significantly higher (*p* < 0.001) than Chinese male and female students. As for BMI, the median BMI of international males was higher (*p* < 0.001) from international female students and Chinese males from Chinese female students. Also, the median BMI of international male and female students were significantly higher (*p* < 0.001) than Chinese males and females. A significant (*p* < 0.001) difference in sleeping time of international and Chinese males, and international and Chinese females were also observed.

### 3.3. Comparison of Nutritional KAP between International and Chinese Students 

**Figure 1:**
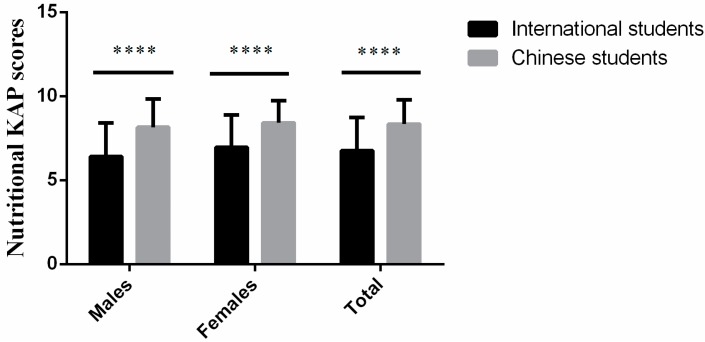
Comparison of nutritional KAP scores between international and Chinese students. Nutritional KAP scores were compared between International students and Chinese students. **** indicates the significant difference (*p* < 0.0001) by Mann Whitney test. International males = 110, Chinese males = 115, International Females = 198, Chinese Females = 278, Total International students = 308 and Total Chinese students = 393.

Figure 1 depicts the comparison of Nutritional KAP scores between international and Chinese students. The KAP scores of Chinese students were significantly higher than international students (*p <* 0.0001). Furthermore, Chinese males and females were higher KAP scores than international males and females respectively (*p <* 0.0001). Also, as a whole, male students were having statistically lower (*p* = 0.003) KAP scores than females students (data not showed). The correct responses about KAP of international and Chinese students have been shown in Supplementary Materials Table S2.

### 3.4. Comparison of Dietary Consumption between International and Chinese Students

**Figure 2:**
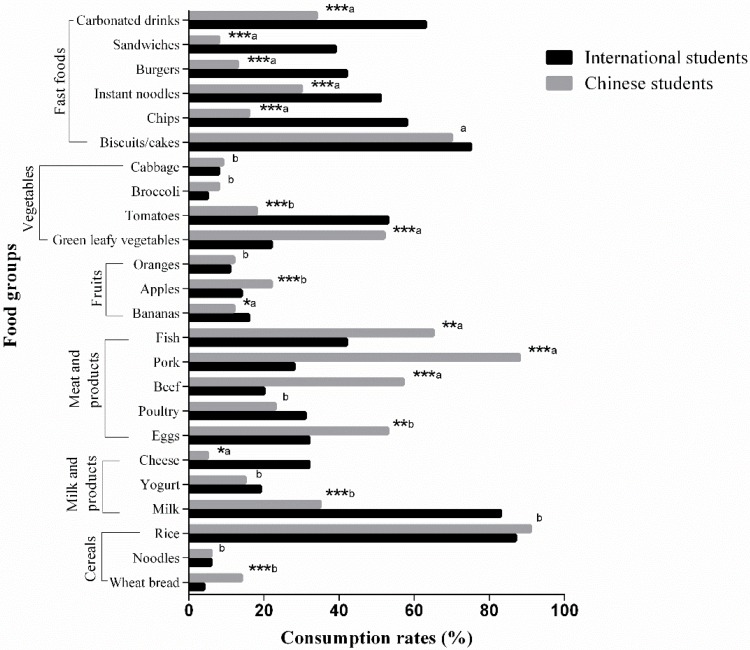
Comparison of dietary intake between international and Chinese students. Dietary intakes between international and Chinese students were on weekly basis. * indicates *p <* 0.05, ** indicates *p <* 0.01, *** indicates *p* < 0.001, a = 1 time/week, b = 7 times/week. *χ*^2^-test was used for comparison.

Figure 2 shows comparison of different food items between international and Chinese students. The consumption of milk on daily basis was significantly (*p* < 0.001) higher in international students. More than 50% of Chinese students were eating significantly (*p* < 0.001) more eggs on daily basis as compare to 32.8% of international students. Daily consumption of poultry was high in international students while beef and pork consumption was higher in Chinese students. Fish eating was significantly (*p* < 0.001) more common on weekly basis in Chinese students. As for green leafy vegetables, the daily percentage was recorded significantly (*p* < 0.001) more in Chinese students. Moreover, fast foods and carbonated drinks consumption were significantly (*p* < 0.001) higher in international students on weekly basis.

### 3.5. Association of Nutritional Status with Determinants

Multivariate models confirmed an independent association of nutritional status based on BMI with sex, educational level, sleeping duration, physical exercise and nutritional KAP scores (Table 3). 

## 4. Discussion

The current research set out to investigate nutritional status, nutritional KAP and dietary consumption in international students with Chinese students.

Nutrition knowledge might be an influential factor for improving nutritional status as it may lead to good dietary attitude and practices, which ultimately boost nutritional status. Students with greater nutritional knowledge adopt good dietary habits. Earlier, a study showed that students having greater nutritional knowledge consume less unhealthy fats and cholesterol [15]. In our study, we found that Chinese students had more nutritional KAP scores as compare to international students. We have been unable to trace any literature regarding comparison of KAP scores between international students and local students. 

The prevalence of overweight and obesity in our study was 12.8% and 5.4% respectively, higher than a previous study in Chinese medical students [23]. The reason may be change in dietary habits and life style of the medical students over the decade. As according to our hypothesis, the prevalence of overweight and obesity in international male and students were higher than Chinese male and female students. Also, BMI for both male and female international students were higher from Chinese male and female students. Similar to our data, a study in Chinese university students showed mean BMI for males (21.5 kg/m^2^) and females (21.9 kg/m^2^) [24]. In contrast to our study, a Chinese cross-sectional study reported 8.3% overweight and 3.0% obesity in international students, and 18.3% of overweight and 6% of obesity in Chinese students [25]. The data showed by the aforementioned study include participants from both medical and non-medical background but the respondents in our study were from medical background. Moreover, the combined prevalence of underweight in both groups was more than 10%. These results were in line with a Serbian study, where the prevalence of underweight in university students was also more than 10%. Because body composition and BMI are associated with physical and emotional wellbeing lead to eating disorder as well as academic pressure and not consumptions of regular meal, which leads to underweight [26]. Another possible reason for underweight in international students may be negative effect of adoptability to new environment. On the other hand, Chinese students are more conscious about their slimness, may lead to high percentages of underweight. Our results were in contrast in terms of underweight with a Chinese study, which showed more percentage of underweight students in international students compared to Chinese students [25].

Increase in fats content and decrease in cardio-respiratory endurance, significantly increases cardiovascular risk. In our study, the mean percent body fats of all female medical students was 30.5%, higher from an earlier study in medical female students, where the mean percent body fats were 24.4% [27]. Also, in our study, international female students were having higher percent body fats (33.1%) compared to Chinese female students (27.9%). Similarly, we found that the mean body fats % of male students were 19.5%. Inline to our study, a Polish study reported 19.3% of percent body fats in male medical students [28]. Furthermore, we found that the body fats percentage of male international students were alleviated from normal ranges and were significantly higher than Chinese male students. The reason might be differences in life style and dietary habits between the two groups. 

As for the daily numbers of hours sleep, we found that Chinese male and female students were found to have good habits of complete sleep (more than 6 h) as compared to international males and females students. Sleep of less than 6 hours is considered as short sleep [29]. A study found that both short and long time spent in bed and poor sleep quality is having positive relationship with overweight and obesity [30]. Short sleep might be another reason for high fats percentages and BMI in international students.

International students may have freedom to adopt new life style choices e.g., eating habits either good or bad, sleeping routines, exercise, etc. [25]. Dietary habits can be a factor for determining nutritional and health status [17]. Consumptions of balanced and healthy diet containing variety of food are accepted [31]. We found that consumption of milk was higher in international students. Eggs, one of the most nutritious foods, with significant proteins, minerals and vitamins, could be included in diet for all ages, and especially for those at risk of low nutrients intakes. Also, daily consumption can help in improvement of dietary habits and nutritional status [32]. Due to importance of egg consumption in young age, we investigated that daily egg consumption was high in Chinese students (53.4%) as compared to international students. These figures are not satisfactory in young age people, who need more nutrients for their nourishment. Another good dietary habit is eating of fish in young people. Weekly consumption of fish was high in Chinese students. Similar to our study, a study in Poland stated that 46% of university students either never eat fish or eat occasionally [33]. Also, meat (beef and pork) consumption (once/week) was high in Chinese students. A Serbian study documented that 80.1% of students consumed meat twice a week [26]. High consumptions of fruits and vegetables have been associated with lower risk of all causes of mortality [34]. However in our study, this consumption was low in both international and Chinese students group. Previously, a study reported low consumption of vegetables and fruits in medical university students [35]. In vegetables, we found that the consumptions of green leafy vegetables on daily basis were higher in Chinese students as compare to international students. Fast food, a high energy-dense diet, low in fiber, and poor in micronutrients, associated with unhealthy weight gain and obesity [36]. In our study, most students consumed fast food on weekly basis, but this percentage was found more in international students. A study in medical students showed that 60% of the medical students consume fast food from weekly basis to monthly basis [36]. Another study reported that in US adults 57% of 18–29 years eat fast food on weekly basis and 33% eat on monthly basis [37]. Among the university students, an unhealthy dietary habit was consumptions of carbonated drinks. Weekly consumption of carbonated drinks was recorded 63.0% in international students, almost double of Chinese students. It is well documented that consumption of carbonated drinks is high in young age group (19–39 years) [38]. Consumption of sugar sweet beverages has been associated with weight gain, overweight, obesity and colonization of oral cavity by fungi [39,40,41]. A study in university students showed that most (80%) of the students take carbonated drinks more than four times a week [7]. A high percentage of fast foods and carbonated drinks consumption may be one of the reasons of high prevalence of overweight and obesity as well as high percent body fats in international students. 

Nutritional status based on BMI was associated with different determinants. In the present study, we found that BMI increases with increase in educational levels. These findings were in contrast with previous finding which states an inverse association between education and BMI [42]. The reason may be the difference between the target populations of the two studies. Also, possibly university students adopt more sedentary life style with increase in an educational level. Inline to our findings, Deng et al. also reported the high prevalence of overweight and obesity in students with high educational levels [43]. We found a negative association of sleeping duration with BMI, these results was consistent with an earlier study [44]. In our study, there was negative association of BMI with physical exercise. Earlier, a study found that lower levels of physical activity leads to increase risk of excess weight [45]. Our finding that nutritional KAP scores had an inverse association with BMI was in accord to a previous study, where negative association of nutritional knowledge with BMI has been reported [46]. Up to our best knowledge, this is the first study, which found an inverse association between nutritional KAP scores and BMI in medical students in China. 

Certain limitations of our study should be account. Data regarding demography, KAP and FFQ were self-reported, especially recalling frequency for each food over one month may have bias. Another limitation of the study is its observational content, only focus on the current status of both international and Chinese students. Nevertheless, the current study is the first attempt to compare nutritional status, KAP and dietary intake between international and Chinese students.

## 5. Conclusions

Our findings highlight that international students have lower nutritional KAP scores, life style, nutritional status, and poor eating practices as compare to local Chinese students. Keeping these highlights in mind, our findings suggest proper nutrition education and knowledge about healthy food practices, that beneficial for improving nutritional and healthy behaviors in international students.

## Figures and Tables

**Table 1:** Baseline characteristics of international and Chinese students.

Variables		International	Chinese	*p* Value
Age (years)		20.7 ± 2.3	19.8 ± 1.3	<0.001 ^a^
Sex	Male	110 (35.7)	115 (29.3)	0.069 ^b^
	Female	198 (64.3)	278 (70.7)	
Education	1st year	89 (28.9)	58 (14.8)	<0.001 ^b^
	2nd year	79 (25.6)	164 (41.7)	
	3rd year	79 (25.6)	132 (33.6)	
	4th year	61 (19.8)	39 (10.0)	
Financial status	Not satisfactory	3 (1.0)	12 (3.1)	0.015 ^b^
	Satisfactory	225 (72.4)	307 (78.1)	
	Very good	80 (26)	74 (18.8)	
BMI (Kg/m^2^)	<18.5	34 (10.9)	65 (16.5)	<0.001 ^b^
	18.5–24.9	196 (63.2)	289 (73.5)	
	25.0–29.9	50 (16.1)	30 (7.6)	
	>30	28 (9.0)	9 (2.3)	
Sleeping time	<4 h/d	5 (1.6)	3 (0.8)	<0.001 ^b^
	4–6 h/d	103 (33.4)	19 (4.8)	
	6–8 h/d	182 (59.1)	336 (85.5)	
	>8 h/d	18 (5.8)	35 (8.9)	
Physical exercise	≥5 days/week	61 (19.8)	80 (20.4)	0.195 ^b^
	3–4 days/week	93 (30.2)	96 (24.4)	
	1–2 days/week	98 (31.8)	152 (38.7)	
	No exercise	56 (18.2)	65 (16.5)	
Total		308 (43.9)	393 (56.1)	

^a^ Independent *t* test, ^b^ Chi square test. Data are expressed as Mean ± SD, and n (%). h/d = hours/day.

**Table 2:** Comparison of anthropometric and life style variables between males and females in international and Chinese students.

Variables		International Students			Chinese Students				
		Male	Female	*p* Value	Male	Female	*p* Value	P1	P2
Percent body fats		21.1 ± 7.5	33.1 ± 6.9	0.001 ^a^	18.0 ± 6.2	27.9 ± 4.8	<0.001 ^a^	0.001 ^a^	<0.001 ^a^
BMI (kg/m^2^)		22.8 (5.2)	21.5 (4.5)	0.02 ^b^	21.0 (3.8)	20.5 (3.3)	<0.001 ^b^	<0.001 ^b^	<0.001 ^b^
Sleeping time	<4 h/d	3.6	0.5	0.079 ^c^	0.0	1.1	0.227 ^c^	<0.001 ^c^	<0.001 ^c^
	4–6 h/d	34.5	32.8		7.8	3.6			
	6–8 h/d	53.3	62.1		83.5	86.3			
	>8 h/d	8.2	4.5		8.7	9.0			

^a^ Independent *t* test, ^b^ Mann Whitney test, ^c^ Chi square test, P1 = *p* valve after comparison of international male with Chinese male students, P2 = *p* valve after comparison of international male with Chinese female students. Data are expressed as Mean ± SD, Median (IQR), and %. h/d = hours/day.

**Table 3:** Association of determinants with nutritional status.

Determinants of BMI	Univariate Analysis		Multivariate Analysis	
	*ß*	*p* Value	*ß*	*p* Value
Sex	−1.035	0.001	−0.724	0.022
Age	0.262	0.001	0.177	0.052
Educational level	0.426	0.005	0.365	0.036
Financial status	0.121	0.694		
Sleeping duration	−0.752	0.007	−0.562	0.047
Physical exercise	−0.247	0.094	−0.311	0.036
Nutritional KAP scores	−0.208	0.008	−0.203	0.012

## Supplementary Materials

The following are available online at http://www.mdpi.com/1660-4601/15/9/1910/s1, Table S1: Description of students according to their country, Table S2: Responses of international and Chinese students.

## References

[1] De Vriendt T., Matthys C., Verbeke W., Pynaert I., De Henauw S. (2009). Determinants of nutrition knowledge in young and middle-aged Belgian women and the association with their dietary behaviour. Appetite.

[2] Perlstein R., McCoombe S., Shaw C., Nowson C. (2016). Medical students’ perceptions regarding the importance of nutritional knowledge and their confidence in providing competent nutrition practice. Public Health.

[3] Edwards J.S., Hartwell H.L., Brown L. (2010). Changes in food neophobia and dietary habits of international students. J. Hum. Nutr. Diet..

[4] Worsley A. (2002). Nutrition knowledge and food consumption: Can nutrition knowledge change food behaviour?. Asia Pac. J. Clin. Nutr..

[5] Kearney J.M., Gibney M.J., Livingstone B.E., Robson P.J., Kiely M., Harrington K. (2001). Attitudes towards and beliefs about nutrition and health among a random sample of adults in the Republic of Ireland and Northern Ireland. Public Health Nutr..

[6] ZarrazquinArizaga I., Atucha A.F., Kortajarena M., Torres-Unda J., Irazusta A., Ruiz-Litago F., Irazusta J., Casis L., Fraile-Bermúdez A.B. (2018). Associations of Anthropometric Characteristics, Dietary Habits, and Aerobic Capacity With Cardiovascular Risk Factors of Health-Science Students. Biol. Res. Nurs..

[7] Musaiger A.O., Awadhalla M.S., Al-Mannai M., AlSawad M., Asokan G.V. (2017). Dietary habits and sedentary behaviors among health science university students in Bahrain. Int. J. Adolesc. Med. Health.

[8] El Ansari W., Stock C., Mikolajczyk R.T. (2012). Relationships between food consumption and living arrangements among university students in four European countries—A cross-sectional study. Nutr. J..

[9] Perez-Cueto F., Verbeke W., Lachat C., Remaut-De Winter A.M. (2009). Changes in dietary habits following temporal migration. The case of international students in Belgium. Appetite.

[10] Lee J., Gao R.R., Kim J.H. (2015). Acculturation and changes in dietary behavior and anthropometric measures among Chinese international students in South Korea. Nutr. Res. Pract..

[11] Almohanna A., Conforti F., Eigel W., Barbeau W. (2015). Impact of Dietary Acculturation on the Food Habits, Weight, Blood Pressure, and Fasting Blood Glucose Levels of International College Students. J. Am. Coll. Health.

[12] Department of Education and Training, Austrailian Government (2016). China-Outbound and Inbound International Students. https://internationaleducation.gov.au/research/Research-Snapshots/Documents/China_outbound%20and%20inbound%20tertiary%20students.pdf.

[13] Zhang J., Qi Q., Delprino R.P. (2017). Psychological health among Chinese college students: A rural/urban comparison. J. Child Adolesc. Ment. Health.

[14] Pi-Sunyer X.F. (1998). Clinical Guidelines on the Identification, Evaluation, and Treatment of Overweight and Obesity in Adults—The Evidence Report. Obes. Res..

[15] Yahia N., Brown C.A., Rapley M., Chung M. (2016). Level of nutrition knowledge and its association with fat consumption among college students. BMC Public Health.

[16] Amiri P., Asghari G., Sadrosadat H., Karimi M., Amouzegar A., Mirmiran P., Azizi F. (2017). Psychometric Properties of a Developed Questionnaire to Assess Knowledge, Attitude and Practice Regarding Vitamin D (D-KAP-38). Nutrients.

[17] Son S., Ro Y., Hyun H., Lee H., Song K. (2014). A comparative study on dietary behavior, nutritional knowledge and life stress between Korean and Chinese female high school students. Nutr. Res. Pract..

[18] Gautreau S., Monsen E.R. (1979). Priorities of nutritional concepts assigned by health professionals and students. J. Med. Educ..

[19] Moss S., Prosser H., Costello H., Simpson N., Patel P., Rowe S., Turner S., Hatton C. (1998). Reliability and validity of the PAS-ADD Checklist for detecting psychiatric disorders in adults with intellectual disability. J. Intellect. Disabil. Res..

[20] El Ansari W., Berg-Beckhoff G. (2015). Nutritional Correlates of Perceived Stress among University Students in Egypt. Int. J. Environ. Res. Public Health.

[21] Lupi S., Bagordo F., Stefanati A., Grassi T., Piccinni L., Bergamini M., De Donno A. (2015). Assessment of lifestyle and eating habits among undergraduate students in northern Italy. Annali dell’Istituto Superiore di Sanita.

[22] Weber K.S., Buyken A.E., Nowotny B., Strassburger K., Simon M.C., Pacini G., Szendroedi J., Mussig K., Roden M., GDS Group (2016). The Impact of Dietary Factors on Glycemic Control, Insulin Sensitivity and Secretion in the First Years after Diagnosis of Diabetes. Exp. Clin. Endocrinol. Diabetes.

[23] Sakamaki R., Toyama K., Amamoto R., Liu C.J., Shinfuku N. (2005). Nutritional knowledge, food habits and health attitude of Chinese university students—A cross sectional study. Nutr. J..

[24] Jingya B., Ye H., Jing W., Xi H., Tao H. (2013). Quantitative analysis and comparison of BMI among Han, Tibetan, and Uygur university students in Northwest China. Sci. World J..

[25] Lolokote S., Hidru T.H., Li X. (2017). Do socio-cultural factors influence college students’ self-rated health status and health-promoting lifestyles? A cross-sectional multicenter study in Dalian, China. BMC Public Health.

[26] Gazibara T., KisicTepavcevic D.B., Popovic A., Pekmezovic T. (2013). Eating habits and body-weights of students of the University of Belgrade, Serbia: A cross-sectional study. J. Health Popul. Nutr..

[27] Kiss K., Meszaros Z., Mavroudes M., Szmodis M.B., Zsidegh M., Ng N., Meszaros J. (2009). Fitness and nutritional status of female medical university students. Acta Physiol. Hung..

[28] Grygiel-Gorniak B., Tomczak A., Krulikowska N., Przyslawski J., Seraszek-Jaros A., Kaczmarek E. (2016). Physical activity, nutritional status, and dietary habits of students of a medical university. Sport Sci. Health.

[29] Van Lee L., Chia A.R., Loy S.L., Colega M., Tham E.K.H., Cai S., Yap F., Godfrey K.M., Teoh O.H., Goh D. (2017). Sleep and Dietary Patterns in Pregnancy: Findings from the GUSTO Cohort. Int. J. Environ. Res. Public Health.

[30] Kristicevic T., Stefan L., Sporis G. (2018). The Associations between Sleep Duration and Sleep Quality with Body-Mass Index in a Large Sample of Young Adults. Int. J. Environ. Res. Public Health.

[31] (2005). Dietary Guidelines for Americans. https://health.gov/dietaryguidelines/dga2005/document/.

[32] Taguchi C., Kishimoto Y., Suzuki-Sugihara N., Saita E., Usuda M., Wang W., Masuda Y., Kondo K. (2018). Regular egg consumption at breakfast by Japanese woman university students improves daily nutrient intakes: Open-labeled observations. Asia Pac. J. Clin. Nutr..

[33] Kowalcze K., Turyk Z., Drywien M. (2016). Nutrition of students from dietetics profile education in the Siedlce University of Natural Sciences and Humanities compared with students from other academic centres. Roczniki Państwowego Zakładu Higieny.

[34] Wang X., Ouyang Y., Liu J., Zhu M., Zhao G., Bao W., Hu F.B. (2014). Fruit and vegetable consumption and mortality from all causes, cardiovascular disease, and cancer: Systematic review and dose-response meta-analysis of prospective cohort studies. BMJ.

[35] Nola I.A., Jelinic J.D., Matanic D., Pucarin-Cvetkovic J., Bergman Markovic B., Senta A. (2010). Differences in eating and lifestyle habits between first- and sixth-year medical students from Zagreb. Coll. Antropol..

[36] Bergeron N., Al-Saiegh S., Ip E.J. (2017). An Analysis of California Pharmacy and Medical Students’ Dietary and Lifestyle Practices. Am. J. Pharm. Educ..

[37] Dugan A. (2013). Fast Food Still Major Part of U.S. Diet. http://news.gallup.com/poll/163868/fast-food-major-part-diet.aspx.

[38] Nergiz-Unal R., Akal Yildiz E., Samur G., Besler H.T., Rakicioglu N. (2017). Trends in fluid consumption and beverage choices among adults reveal preferences for ayran and black tea in central Turkey. Nutr. Diet..

[39] Malik V.S., Schulze M.B., Hu F.B. (2006). Intake of sugar-sweetened beverages and weight gain: A systematic review. Am. J. Clin. Nutr..

[40] Lim L., Banwell C., Bain C., Banks E., Seubsman S.A., Kelly M., Yiengprugsawan V., Sleigh A. (2014). Sugar sweetened beverages and weight gain over 4 years in a Thai national cohort—A prospective analysis. PLoS ONE.

[41] Góralska K., Klimczak A., Rachubinski P., Jaglowska A., Kwapiszewska A. (2015). Consumption of sweetened beverages as a risk factor of colonization of oral cavity by fungi—Eating habits of university students. Ann. Parasitol..

[42] Hermann S., Rohrmann S., Linseisen J., May A.M., Kunst A., Besson H., Romaguera D., Travier N., Tormo M.J., Molina E. (2011). The association of education with body mass index and waist circumference in the EPIC-PANACEA study. BMC Public Health.

[43] Deng X., Castelli D., Castro-Pinero J., Guan H. (2011). University Students Meeting the Recommended Standards of Physical Activity and Body Mass Index. ICHPER-SD J. Res..

[44] Kanikowska D., Sikorska D., Kuczynska B., Grzymislawski M., Breborowicz A., Witowski J. (2017). Do medical students adhere to advice regarding a healthy lifestyle? A pilot study of BMI and some aspects of lifestyle in medical students in Poland. Adv. Clin. Exp. Med..

[45] Martinez-Moya M., Navarrete-Munoz E.M., Garcia de la Hera M., Gimenez-Monzo D., Gonzalez-Palacios S., Valera-Gran D., Sempere-Orts M., Vioque J. (2014). Association between hours of television watched, physical activity, sleep and excess weight among young adults. Gaceta Sanitaria.

[46] Valmorbida J.L., Goulart M.R., Busnello F.M., Pellanda L.C. (2017). Nutritional knowledge and body mass index: A cross-sectional study. Rev. Assoc. Med. Bras..

