# Supplementary material for: A Comparative Study of Nutritional Status, Knowledge Attitude and Practices (KAP) and Dietary Intake between International and Chinese Students in Nanjing, China
Source: Int J Environ Res Public Health. 2018 Sep 3;15(9):1910. doi: 10.3390/ijerph15091910 (PMC6165259; doi:10.3390/ijerph15091910)
Supplement: Supplementary file 1 [file ijerph-15-01910-s001.pdf]

Supplementary Material

A Comparative Study of Nutritional Status, Knowledge Attitude and Practices (KAP) and Dietary Intake between International and Chinese Students in Nanjing, China

Ijaz ul Haq [1], Zahula Mariyam [1], Min Li [2], Xiaojia Huang [2], Pan Jiang [1], Falak Zeb [1], Xiaoyue Wu [1], Qing Feng [1,*] and Ming Zhou [1,*]

[1] Department of Nutrition and Food Hygiene, Nanjing Medical University, Nanjing 211166, Jiangsu Province, China; ijaz@njmu.edu.cn (I.u.H.); mariyamzahul@gmail.com (Z.M.); jppanpan@njmu.edu.cn (P.J.); falak106@gmail.com (F.Z.); xiaoyuewu@njmu.edu.cn (X.W.)

[2] School of International Education, Nanjing Medical University, Nanjing 211166, China; minli@njmu.edu.cn (M.L.); shawn2006@163.com (X.H.)

* Correspondence: qingfeng@njmu.edu.cn (Q.F.); mzhou78@163.com (M.Z.); Tel.: +86-25-86868412 (Q.F.)

Table S1. Description of students according to their country.

	Country	N	%
International students	India	112	16.0
	Sri Lanka	88	12.6
	Thailand	46	6.6
	USA	3	.4
	Ghana	19	2.7
	Mauritius	9	1.3
	Indonesia	13	1.9
	Others countries	18	2.6
Chinese students	China	393	56.1
	Total	701	100.0

Table S2. Responses of international and Chinese students.

KAP questions	Correct responses	Rate (%)	
		International students	Chinese students
Knowledge			
Nutritional value of food depends on its cooking pattern	T	75.5	66.6
Nutritious food means the food has high calories	F	69.4	93.9
All men and women require the same amount of calories	F	80.0	87.6
A balanced diet which is important for life contain adequate energy and nutrients required	T	89.7	86.5
If a food has a lot of proteins and lipids, there is no need to consume food with carbohydrates like rice and wheat	F	70.0	84.0
We need to use lipids even if someone is obese	T	44.8	93.7
Attitude			
I believe that unhealthy eating habits causes obesity	Agree	81.6	98.0

Table S2. *Cont.*

I believe that high intake of sugar-sweetened beverages leads to unhealthy weight gain	Agree	87.1	95.7
Practice			
Calculation of body mass index (BMI)	Practicing	54.2	73.4
Taking of breakfast (7day/week)	Practicing	25.5	51.5

T=true, F=false
